# Severity Grading, Risk Factors, and Prediction Model of Complications After Epilepsy Surgery: A Large-Scale and Retrospective Study

**DOI:** 10.3389/fneur.2021.722478

**Published:** 2021-10-07

**Authors:** Yong Liu, Hao Wu, Huanfa Li, Shan Dong, Xiaofang Liu, Kuo Li, Changwang Du, Qiang Meng, Hua Zhang

**Affiliations:** ^1^Department of Neurosurgery, The First Affiliated Hospital of Xi'an Jiaotong University, Xi'an, China; ^2^Center of Brain Science, The First Affiliated Hospital of Xi'an Jiaotong University, Xi'an, China; ^3^Center for Mitochondrial Biology and Medicine, The Key Laboratory of Biomedical Information Engineering of Ministry of Education, School of Life Science and Technology, Xi'an Jiaotong University, Xi'an, China; ^4^Clinical Research Center for Refractory Epilepsy of Shaanxi Province, The First Affiliated Hospital of Xi'an Jiaotong University, Xi'an, China

**Keywords:** complication, grading, prediction model, refractory epilepsy, risk factor, surgery

## Abstract

**Purpose:** To report complications after epilepsy surgery, grade the severity of complications, investigate risk factors, and develop a nomogram for risk prediction of complications.

**Methods:** Patients with epilepsy surgery performed by a single surgeon at a single center between October 1, 2003 and April 30, 2019 were retrospectively analyzed. Study outcomes included severity grading of complications occurring during the 3-month period after surgery, risk factors, and a prediction model of these complications. Multivariable logistic regression analysis was used to calculate odds ratio and 95% confidence interval to identify risk factors.

**Results:** In total, 2,026 surgical procedures were eligible. There were 380 patients with mild complications, 23 with moderate complications, and 82 with severe complications. Being male (odds ratio 1.29, 95% confidence interval 1.02–1.64), age at surgery (>40 years: 2.58, 1.55–4.31; ≤ 40: 2.25, 1.39–3.65; ≤ 30: 1.83, 1.18–2.84; ≤ 20: 1.71, 1.11–2.63), intracranial hemorrhage in infancy (2.28, 1.14–4.57), serial number of surgery ( ≤ 1,000: 1.41, 1.01–1.97; ≤ 1,500: 1.63, 1.18–2.25), type of surgical procedure (extratemporal resections: 2.04, 1.55–2.70; extratemporal plus temporal resections: 2.56, 1.80–3.65), surgery duration (>6 h: 1.94, 1.25–3.00; ≤ 6: 1.92, 1.39–2.65), and acute postoperative seizure (1.44, 1.06–1.97) were independent risk factors of complications. A nomogram including age at surgery, type of surgical procedure, and surgery duration was developed to predict the probability of complications.

**Conclusions:** Although epilepsy surgery has a potential adverse effect on the patients, most complications are mild and severe complications are few. Risk factors should be considered during the perioperative period. Patients with the above risk factors should be closely monitored to identify and treat complications timely. The prediction model is very useful for surgeons to improve postoperative management.

## Highlights

- This is the first large-scale study including 2,026 surgical procedures for refractory epilepsy in the last two decades.
- It is the first time that the severity of complications after epilepsy surgery is graded based on the therapeutic regimen. Most complications are mild (380/2,026, 18.8%). Surgical mortality is 0.1%.
- We comprehensively identify the risk factors of complications, providing robust evidence based on the large sample.
- We firstly develop a nomogram for individualized prediction of the probability of complications. Our model is useful for surgeons to identify high-risk patients and enhance the postoperative management for decreasing and avoiding the incidence of complications.

## Introduction

Epilepsy, involving a persistent predisposition to seizure, is one of the most common chronic neurological disorders, affecting more than 65 million people worldwide (1, 2). Epilepsy not only negatively impacts patients' education, employment, and social contact, but also imposes a serious burden on patients' families and on society. Despite the decrease in the disease burden from 1990 to 2016, epilepsy is still an important cause of disability and mortality (3), making it a global public health issue.

Furthermore, about 40% of patients respond poorly to the first two antiepileptic drugs administered and have medically refractory epilepsy (4). Epilepsy surgery is effective for refractory epilepsy, particularly focal epilepsy (5, 6), but is still underutilized worldwide. In the United States, the annual percentage of surgical procedures for refractory epilepsy was low (range: 0.35–0.63%) from 2003 to 2012 (7). Moreover, the number of surgical procedures for mesial temporal sclerosis (the most common type of refractory epilepsy) declined by more than half from 2006 to 2010. Fear associated with the risks of invasive procedures may be the reason for the cautious attitude toward epilepsy surgery (8, 9). Therefore, the risks of epilepsy surgery in the modern age need to be evaluated thoroughly and precisely to improve epilepsy surgery outcomes.

The safety of epilepsy surgery has been confirmed in several studies (10–16). From 1980 to 2012, neurological deficits following epilepsy surgery decreased with time, from 41.8 to 5.2% in temporal resections and from 30.2 to 19.5% in extratemporal resections (17). However, studies on this topic with large sample sizes (>500 patients) were either multicenter (11, 13, 15) or covered a long study period (10, 14). In addition, high-resolution magnetic resonance imaging (MRI) was not used in the early stage in these studies. Differences in medical environment among epilepsy centers and advancements in presurgical evaluations and surgical techniques over time may have caused heterogeneity and biases, thereby limiting the quality of these studies. Over the past two decades, there was no large-scale studies on post-epilepsy surgery complications performed at a single center. Moreover, surgery-related complications are seldom graded according to severity. Especially, the risk factors for these complications remain unclear.

Understanding the incidence and severity of complications after epilepsy surgery and the associated risk factors is beneficial, allowing physicians to provide patients with adequate surgical advice, and allowing patients to make rational decisions regarding epilepsy surgery. Furthermore, this information may help in the prevention of postoperative complications and improve our understanding of the procedures. Therefore, we reported the incidence of complications occurring in a 3-month period after epilepsy surgery was performed by a single neurosurgeon at a single center, identified the associated risk factors, and developed a nomogram for individually predicting the probability of complications.

## Methods

### Patients

This was a single-center, large-scale, and retrospective study. Patient data were retrospectively collected at the single epilepsy center of the tertiary teaching hospital from October 1, 2003, to April 30, 2019. Inclusion criteria included: (1) men or women without age limits; (2) medically refractory epilepsy defined by the International League Against Epilepsy (18); (3) epilepsy surgery performed by a single neurosurgeon, Dr. H.Z.; (4) surgical procedure performed *via* craniotomy; (5) signed informed consent; and (6) good compliance, for at least a 3-month follow-up period after surgery. Exclusion criteria included: (1) drug-responsive epilepsy, seizure freedom with current drugs in the past year, (2) pseudoseizures, (3) significant comorbidities including progressive neurological disorders, active psychosis, and drug abuse; (4) neuromodulation therapy; and (5) poor compliance and inadequate follow-up.

### Presurgical Evaluations and Surgical Procedures

Presurgical evaluations included medical history, seizure semiology, electroencephalography (EEG), imaging examination, and other ancillary investigations (19). Long-term (at least 24-h) video-EEG with scalp electrodes, placed using the international 10–20 system, was monitored for interictal and ictal events. For MRI examination, 3-Tesla brain MRI with T1-weighted, T2-weighted, and FLAIR sequences in 3-dimensional scanning mode was applied after 2005. Enhanced MRI was performed for intracranial space-occupying lesions. Patients for whom it was difficult to localize the seizure onset zone by non-invasive evaluations underwent invasive electrode implantation for intracranial EEG monitoring. Other ancillary investigations included functional MRI, positron emission tomography, and single photon emission computerized tomography.

Surgical procedures were individually designed according to the presurgical evaluation findings. Standard epilepsy surgery procedures were applied (20). Generally, surgical procedures were divided into curative and palliative surgery. Curative surgery included resection and disconnection of the epileptogenic zone. Palliative surgery included corpus callosotomy for bilateral synchronous onset and multiple subpial transections for epileptic foci located in the eloquent cortex. For widespread epileptogenic zones, multiple surgical techniques were combined.

### Outcomes

Complications were defined as any deviation from the normal postoperative course occurring in a 3-month period after surgery (13). Complications included neurologic deficit, cerebral edema, intracranial hemorrhage (ICH), infection, hydrocephalus, subdural effusion, subcutaneous cerebrospinal fluid (CSF) accumulation, and poor wound healing. Visual field defects were inevitable following temporal lobe resection, which most patients were not aware of. If patients complained about visual field defects, then visual field defects were defined as a neurologic complication. Neurologic deficit was classified as either transient (resolving within 3 months) or persistent (lasting more than 3 months) (12, 13).

The method for grading the severity of the complications was slightly modified from those reported in previous studies (21, 22). Complication severity was categorized into four grades based on the therapeutic regimen: grade I, minor complications with conservative treatment; grade II, major complications requiring invasive treatment without general anesthesia; grade III, life-threatening complications requiring invasive treatment under general anesthesia or monitoring in the intensive care unit; grade IV, death. Grades I and II were considered mild and moderate (non-severe), whereas grades III and IV were considered severe. Transient and persistent neurologic deficits were classified as grades I and III, respectively. When more than one complication was present in a patient, the complication with the highest grade was considered.

Risk factors for postoperative complications were analyzed. Potential factors included the preoperative, intra-operative, and postoperative clinical characteristics, such as sex, age at surgery, duration of seizure, previous medical history, pathology, serial number of surgery, invasive electrode implantation, type of surgical procedure, surgery duration, intra-operative blood loss, and acute postoperative seizure (APOS). APOS was defined as seizures occurring during the first postoperative week (23). According to the independent risk factors, a prediction model was established.

### Statistical Analysis

The categorical data were represented as frequencies and percentages and analyzed using the χ^2^ test. Risk factors for complications were determined by using univariate and multivariate logistic regression analyses. Variables with *P* < 0.10 were selected as potential risk factors and included in the multivariate logistic regression analysis. The forward stepwise method was used to select the variables that were eventually included in the model. Odds ratio (OR) and 95% confidence interval (CI) were calculated.

To establish a nomogram for predicting the probability of complications after epilepsy surgery, patients were randomly divided into a development group (70% of all patients) and validation group (30%). Based on the regression coefficients of independent risk factors in the development group, the individualized prediction model was established. We evaluated the prediction model by discrimination and calibration. The discrimination of the prediction model represents its ability to distinguish between patients with complications from those with no complications. We evaluated the discrimination by calculating the area under the curve (AUC) of the receiver operating characteristic (ROC) curve. A prediction model with an AUC of 0.5–0.75 is considered acceptable. The calibration of the prediction model shows the concordance between the predicted and observed probabilities. The Hosmer-Lemeshow test suggests there is good calibration with *P* > 0.05.

Statistical analysis was performed using SPSS software (ver 22.0, USA) and EmpowerStats (www.empowerstats.net; X&Y Solutions Inc., Boston, MA). The logistic regression analyses and calibration were performed by SPSS. The nomogram and ROC curve were plotted by EmpowerStats. Two-tailed analysis with *P* < 0.05 indicated that the difference was statistically significant.

## Results

### Postoperative Complications and Grades

**Figure 1:**
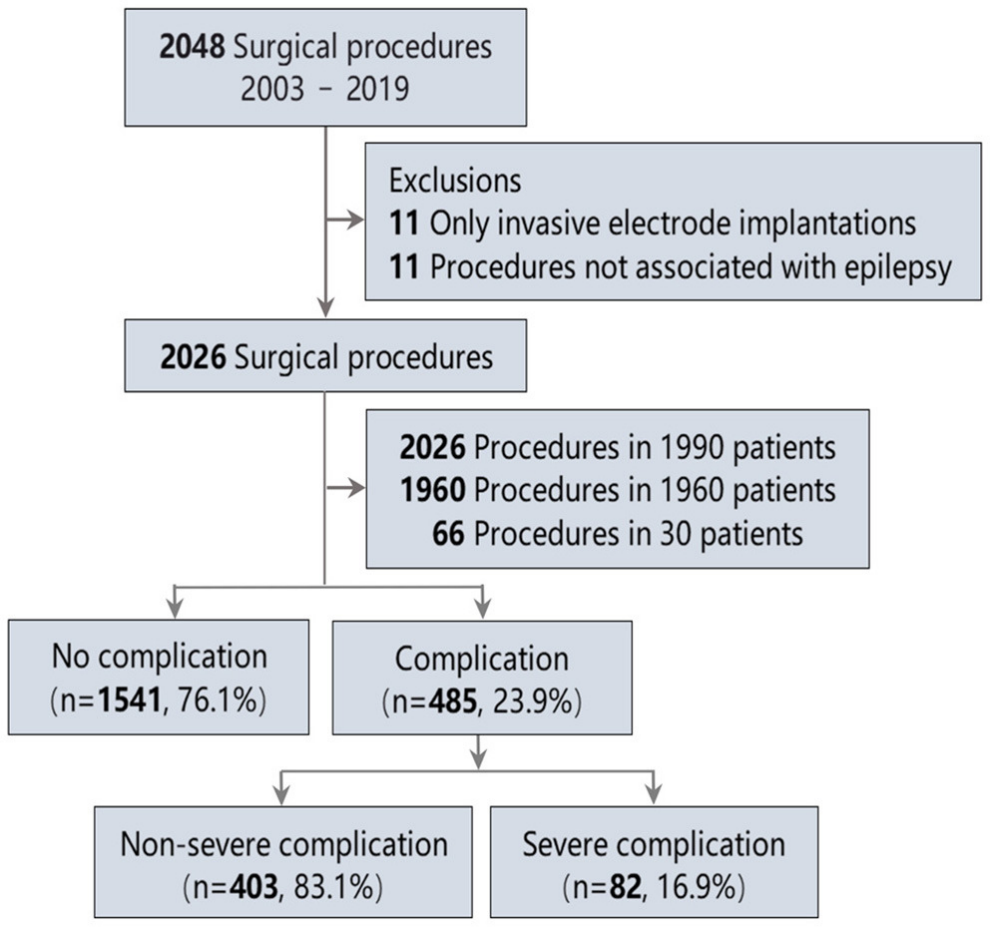
Study diagram of selection process for assigning groups.

Overall, 2,048 inpatient surgical procedures were documented. Finally, 1,990 patients undergoing 2,026 procedures were included (Figure 1). Postoperative complications are shown in Table 1. The most common complication was subcutaneous CSF accumulation (12.2%), followed by neurological deficits (7.3%) and intracranial hemorrhage (3.3%). Neurological deficits were observed in 7.3% of all surgical procedures. Persistent neurological complications were recorded in only 2.3%. Table 2 lists neurological deficits in detail.

**Table 1:** Incidences and grades of complications after epilepsy surgery (608 complications in 485 procedures).

**Characteristics**	**Total (*n*, %)**	**Grades (** ***n*** **, %)**			
		**I**	**II**	**III**	**IV**
Intracranial hemorrhage	66 (3.3)	45 (2.2)	4 (0.2)	16 (0.8)	1 (0.05)
Epidural	38 (1.9)	24 (1.2)	3 (0.1)	11 (0.5)	0 (0.0)
Subdural	5 (0.2)	3 (0.1)	1 (0.05)	0(0.0)	1 (0.05)
Epidural + subdural	1 (0.05)	0 (0.0)	0 (0.0)	1 (0.05)	0 (0.0)
Intraparenchymal	22 (1.1)	18 (0.9)	0 (0.0)	4 (0.2)	0 (0.0)
Brain edema	53 (2.6)	52 (2.6)	0 (0.0)	0 (0.0)	1(0.05)
Hydrocephalus	15 (0.7)	8 (0.4)	3 (0.1)	4 (0.2)	0 (0.0)
Infection	26 (1.3)	10 (0.5)	3 (0.1)	13 (0.6)	0 (0.0)
CNS	11 (0.5)	10 (0.5)	1 (0.05)	0 (0.0)	0 (0.0)
Wound	9 (0.4)	0 (0.0)	1 (0.05)	8 (0.4)	0 (0.0)
CNS + wound	4 (0.2)	0 (0.0)	1 (0.05)	3 (0.1)	0 (0.0)
Lung	2 (0.1)	0 (0.0)	0 (0.0)	2 (0.1)	0 (0.0)
Poor wound healing	23 (1.1)	20 (1.0)	3 (0.1)	0 (0.0)	0 (0.0)
Subcutaneous CSF accumulation	248 (12.2)	236 (11.6)	11 (0.5)	1 (0.05)	0 (0.0)
Subdural effusion	12 (0.6)	9 (0.4)	3 (0.1)	0 (0.0)	0 (0.0)
Deep venous thrombosis	5 (0.2)	2 (0.1)	2 (0.1)	1 (0.05)	0 (0.0)
Neurologic deficits	147 (7.3)	101 (5.0)	0 (0.0)	46 (2.3)	0 (0.0)
Transient	101 (5.0)	101 (5.0)	0 (0.0)	0 (0.0)	0 (0.0)
Persistent	46 (2.3)	0 (0.0)	0 (0.0)	46 (2.3)	0 (0.0)
Other complications^a^	13 (0.6)	10 (0.5)	0 (0.0)	2 (0.1)^b^	1 (0.05)^c^
Total	608	493	29	83	3

*CNS, central nervous system; CSF, cerebrospinal fluid.*

a *Other complications included auricular perichondritis in one patient, massive hemorrhage of upper digestive tract in one patient, massive thoracic bleeding following subclavian vein catheterization in one patient, urethral injury in one patient, diabetes insipidus in one patient, cerebral vasospasm in two patients, cerebral infarction in two patients, acute lumbar disc herniation in one patient, severe hyponatremia in one patient, intestinal tympanites in one patient, hyperglycemia in one patient.*

b *Thoracotomy for hemostasis in one patient with massive thoracic bleeding. One patient with massive hemorrhage of upper digestive tract was closely monitored in the intensive care unit.*

c *One patient died of severe hyponatremia*.

**Table 2:** Detailed neurological deficits.

**Neurological**	**Transient**	**Persistent**	**Total**
**deficits**	**(*n* = 101)**	**(*n* = 46)**	**(*n* = 147)**
Paralysis	65	28	93
Hemiparesis	39	15	54
Monoparesis	26	13	39
Paresthesia	12	6	18
Oculomotor paralysis	4	4	8
Dysphasia	38	8	46
Visual field defects	1	2	3
Conscious disturbance	7	3	10

The complication severity grades are presented in Table 1. Among 485 patients with complications, 380 patients (78.4%) had grade I complications; 23 (4.7%), grade II; 79 (16.3%), grade III; and 3 (0.6%), grade IV. The incidences of non-severe and severe complications were 19.9% (403/2,026) and 4% (82/2,026), respectively. Three patients died after surgery (mortality: 0.1%).

### Risk Factors for Complications

Table 3 shows risk factors for complications. In the univariate analysis, sex, age at surgery, previous craniotomy, previous traumatic brain injury, ICH in infancy, pathology, serial number of surgery, invasive electrode implantation, type of surgical procedure, surgery duration, intra-operative blood loss, and APOS were potential risk factors of complications.

**Table 3:** Potential factors for complications.

**Characteristics**	**Total**	**No complications**	**Non-severe complications**	**Severe complications**	***P-*value**
	**(*n* = 2,026, %)**	**(*n* = 1,541, %)**	**(*n* = 403, %)**	**(*n* = 82, %)**	
Sex					0.004
Female	675 (33.3)	543 (35.2)	108 (26.8)	24 (29.3)	
Male	1,351 (66.7)	998 (64.8)	295 (73.2)	58 (70.7)	
Age at surgery, y					0.030
≤ 10	217 (10.7)	180 (11.7)	31 (7.7)	6 (7.3)	
≤ 20	602 (29.7)	458 (29.7)	122 (30.3)	22 (26.8)	
≤ 30	652 (32.2)	499 (32.4)	131 (32.5)	22 (26.8)	
≤ 40	313 (15.4)	231 (15.0)	69 (17.1)	13 (15.9)	
>40	242 (11.9)	173 (11.2)	50 (12.4)	19 (23.2)	
Duration of seizure, y					0.623
≤ 5	838 (41.4)	627 (40.7)	174 (43.2)	37 (45.1)	
≤ 10	449 (22.2)	350 (22.7)	83 (20.6)	16 (19.5)	
≤ 15	320 (15.8)	236 (15.3)	72 (17.9)	12 (14.6)	
>15	419 (20.7)	328 (21.3)	74 (18.4)	17 (20.7)	
Previous craniotomy					0.000
No	1,790 (88.4)	1,390 (90.2)	337 (83.6)	63 (76.8)	
Yes	236 (11.6)	151 (9.8)	66 (16.4)	19 (23.2)	
Previous meningitis/encephalitis					0.462
No	1,875 (92.5)	1,428 (92.7)	374 (92.8)	73 (89.0)	
Yes	151 (7.5)	113 (7.3)	29 (7.2)	9 (11.0)	
Previous traumatic brain injury					0.007
No	1,715 (84.6)	1,326 (86.0)	322 (79.9)	67 (81.7)	
Yes	311 (15.4)	215 (14.0)	81 (20.1)	15 (18.3)	
ICH in infancy					0.012
No	1,987 (98.1)	1,519 (98.6)	388 (96.3)	80 (97.6)	
Yes	39 (1.9)	22 (1.4)	15 (3.7)	2 (2.4)	
Pathology					0.000
No available	604 (29.8)	463 (30.0)	117 (29.0)	24 (29.3)	
Tumor	330 (16.3)	241 (15.6)	64 (15.9)	25 (30.5)	
Vascular malformation	88 (4.3)	65 (4.2)	18 (4.5)	5 (6.1)	
Gliosis	380 (18.8)	263 (17.1)	98 (24.3)	19 (23.2)	
Cortical dysplasia	624 (30.8)	509 (33.0)	106 (26.3)	9 (11.0)	
Serial number of surgery					0.000
≤ 500	500 (24.7)	380 (24.7)	93 (23.1)	27 (32.9)	
≤ 1,000	500 (24.7)	371 (24.1)	110 (27.3)	19 (23.2)	
≤ 1,500	500 (24.7)	355 (23.0)	125 (31.0)	20 (24.4)	
>1,500	526 (26.0)	435 (28.2)	75 (18.6)	16 (19.5)	
Type of surgery					0.933
Palliative surgery	140 (6.9)	106 (6.9)	29 (7.2)	5 (6.1)	
Curative surgery	1,886 (93.1)	1,435 (93.1)	374 (92.8)	77 (93.9)	
Type of surgical procedure					0.000
Temporal resections	871 (43.0)	728 (47.2)	127 (31.5)	16 (19.5)	
Extratemporal resections	880 (43.4)	641 (41.6)	191 (47.4)	48 (58.5)	
Combination	275 (13.6)	172 (11.2)	85 (21.1)	18 (22.0)	
Invasive electrode implantation					0.034
No	1,825 (90.1)	1,403 (91.0)	351 (87.1)	71 (86.6)	
Yes	201 (9.9)	138 (9.0)	52 (12.9)	11 (13.4)	
Surgery duration, h					0.000
≤ 4	789 (38.9)	640 (41.5)	125 (31.0)	24 (29.3)	
≤ 5	696 (34.4)	536 (34.8)	126 (31.3)	34 (41.5)	
≤ 6	383 (18.9)	263 (17.1)	108 (26.8)	12 (14.6)	
>6	158 (7.8)	102 (6.6)	44 (10.9)	12 (14.6)	
Intra-operative blood loss, ml					0.005
≤ 250	576 (28.4)	462 (30.0)	94 (23.3)	20 (24.4)	
≤ 350	565 (27.9)	442 (28.7)	103 (25.6)	20 (24.4)	
≤ 450	421 (20.8)	315 (20.4)	88 (21.8)	18 (22.0)	
>450	464 (22.9)	322 (20.9)	118 (29.3)	24 (29.3)	
Acute postoperative seizure					0.004
No	1,778 (87.8)	1,373 (89.1)	335 (83.1)	70 (85.4)	
Yes	248 (12.2)	168 (10.9)	68 (16.9)	12 (14.6)	

*ICH, intracranial hemorrhage*.

**Figure 2:**
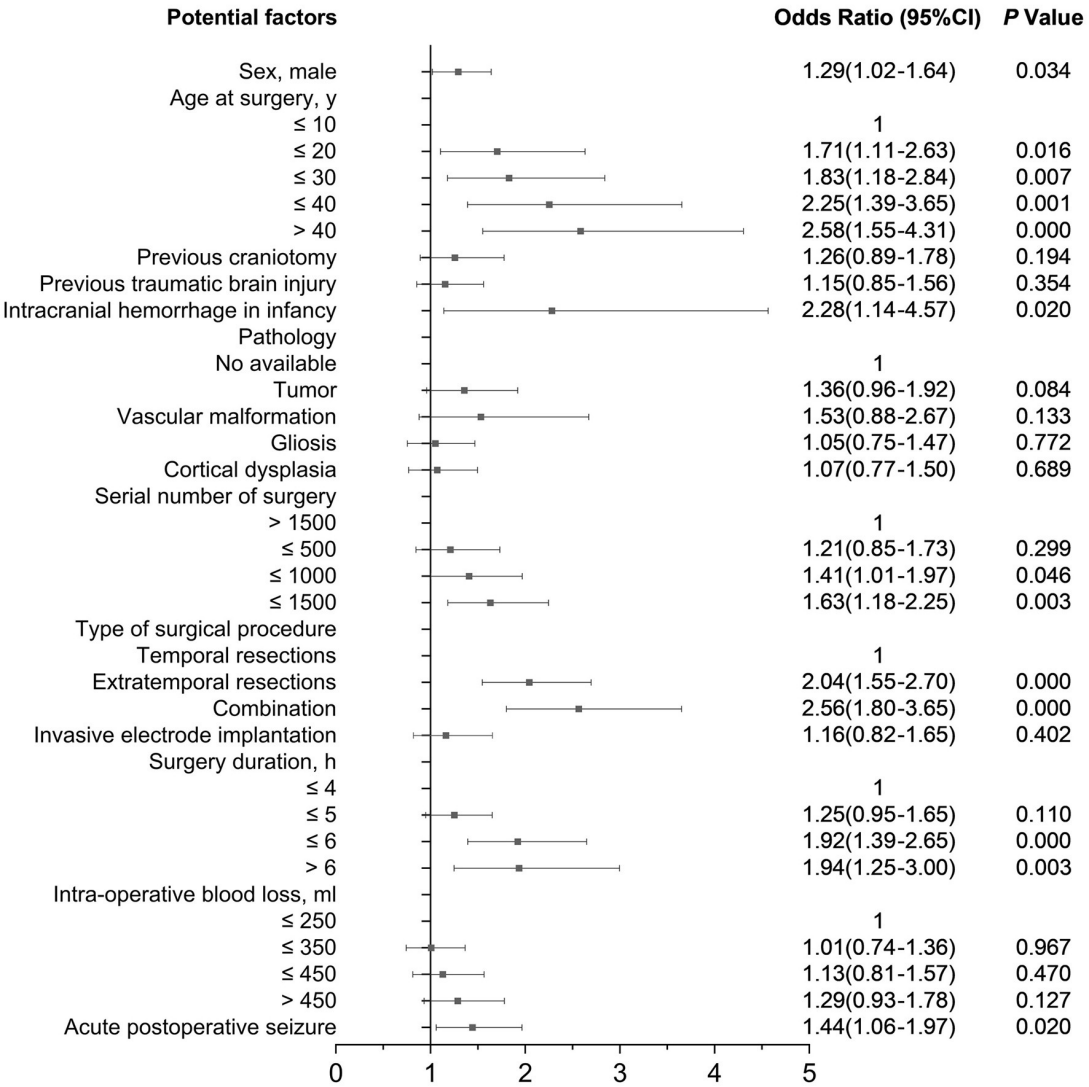
Risk factors for complications in multivariate analysis.

Factors of complications that remain statistically significant in the multivariable analysis are delineated in Figure 2. Risk factors included being male (OR 1.29, 95% CI 1.02–1.64), age at surgery (>40 years: 2.58, 1.55–4.31; ≤ 40: 2.25, 1.39–3.65; ≤ 30: 1.83, 1.18–2.84; ≤ 20: 1.71, 1.11–2.63), ICH in infancy (2.28, 1.14–4.57), serial number of surgery ( ≤ 1,000: 1.41, 1.01–1.97; ≤ 1,500: 1.63, 1.18–2.25), type of surgical procedure (extratemporal resections: 2.04, 1.55–2.70; extratemporal plus temporal resections: 2.56, 1.80–3.65), surgery duration (>6 h: 1.94, 1.25–3.00; ≤ 6: 1.92, 1.39–2.65), and APOS (1.44, 1.06–1.97), which significantly increased the likelihood of postoperative complications.

**Figure 3:**
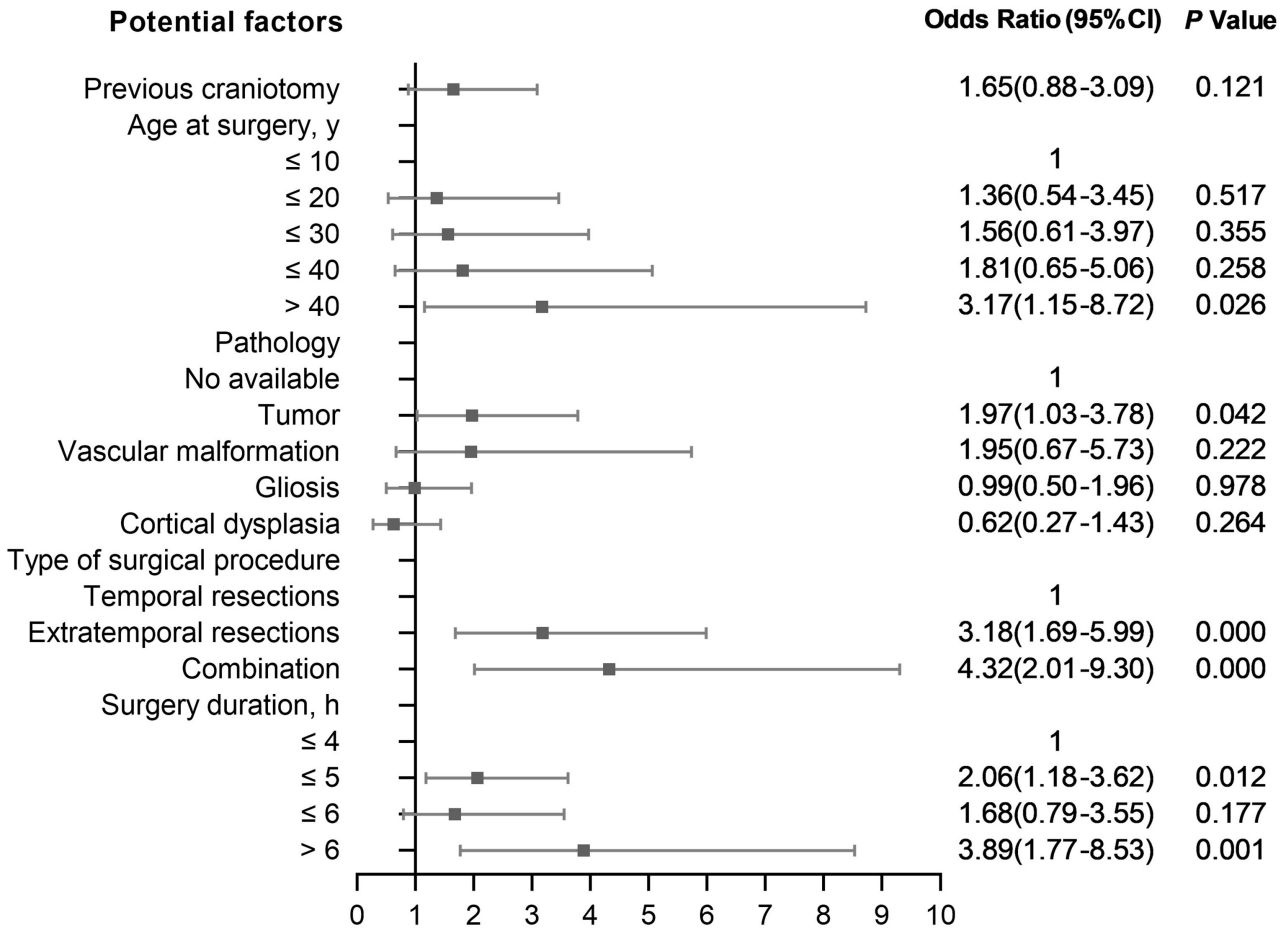
Risk factors for severe complications compared with no complications.

Compared with patients with no complications, factors of severe complications that remain statistically significant are shown in Figure 3. Multivariable analysis found that age at surgery (>40 years: 3.17, 1.15–8.72), tumor (1.97, 1.03–3.78), type of surgical procedure (extratemporal resections: 3.18, 1.69–5.99; extratemporal plus temporal resections: 4.32, 2.01–9.30), surgery duration (>6 h: 3.89, 1.77–8.53; ≤ 5: 2.06, 1.18–3.62) were significantly associated with the incidence of severe complications compared with no complications.

**Figure 4:**
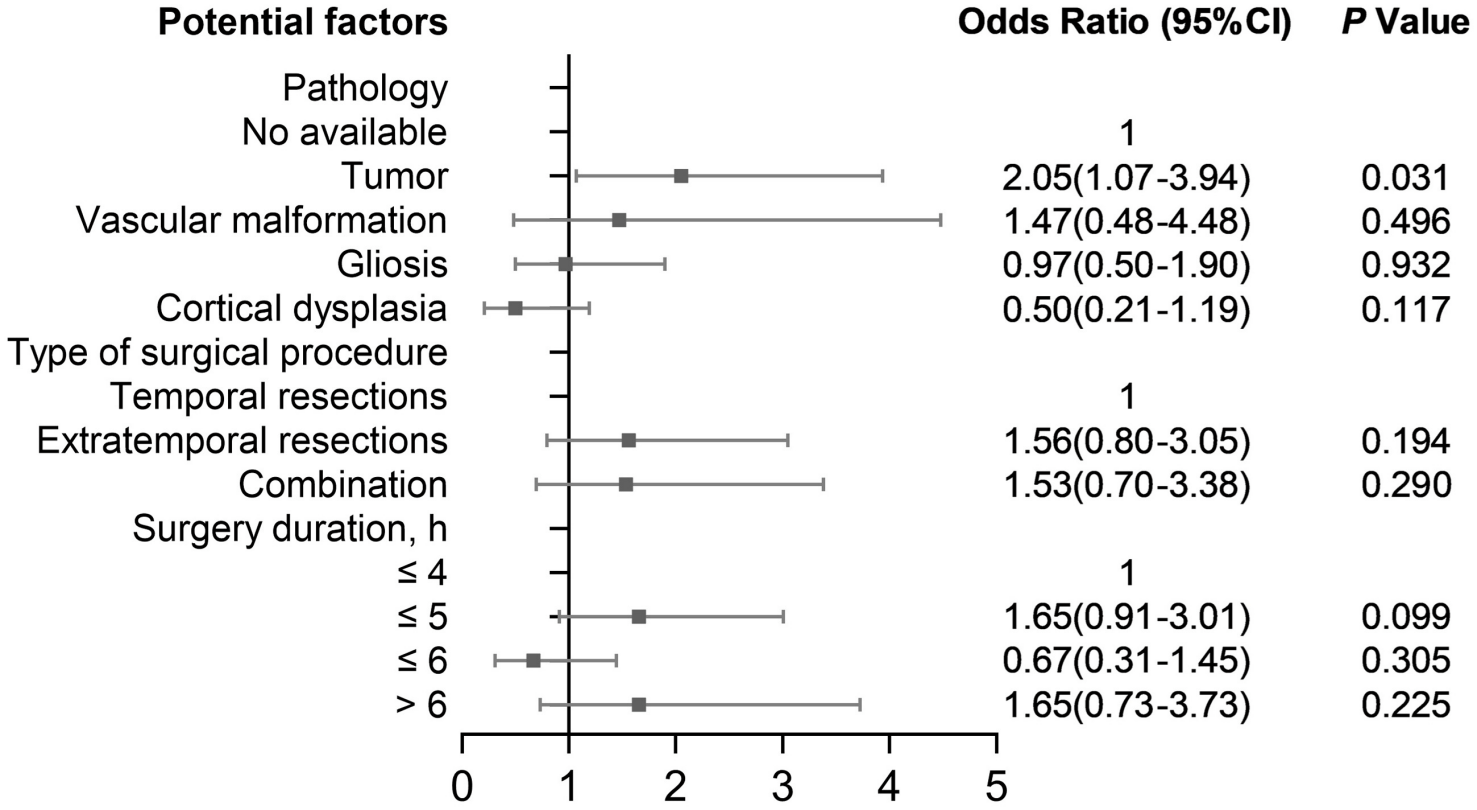
Risk factors for severe complications compared with non-severe complications.

Figure 4 shows risk factors of severe complications compared with patients with non-severe complications. In the multivariable analysis, only having a tumor (2.05, 1.07–3.94) was an independent risk factor of severe complications compared with non-severe complications.

### The Prediction Model of Complications

**Figure 5:**
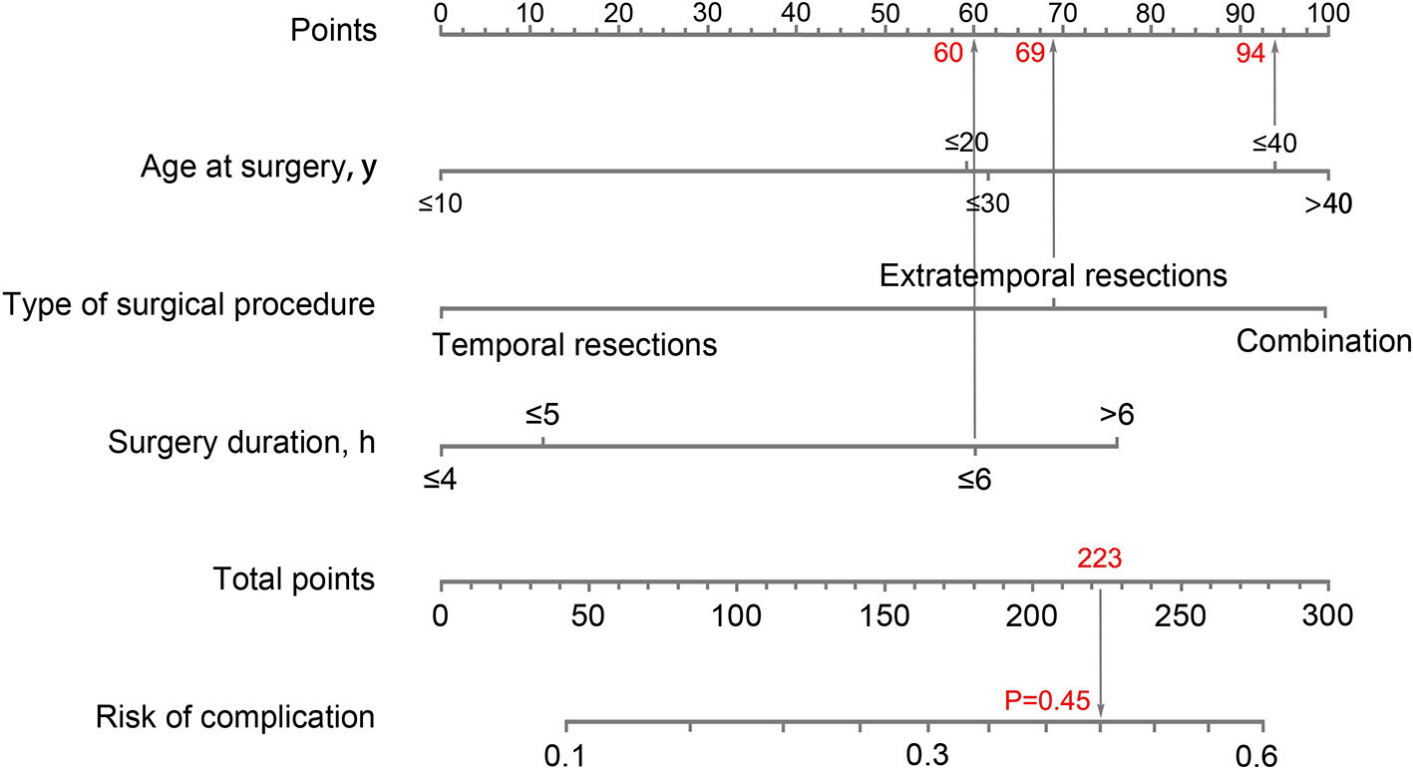
Nomogram to predict the probability of complications. An example of how to use the nomogram: a 33-year-old man received extratemporal resection for refractory epilepsy; the duration of surgical procedure was 6 h; the total points for each risk factor add up to 223; a vertical line was then drawn from 223 on the “Total points” line down to the last line to predict the probability of complications (45%).

We developed a nomogram of individually predicting the probability of complications (Figure 5). Patients were divided into a development group (*n* = 1,420) and validation group (*n* = 606). Based on the logistic multivariate regression analysis, three factors of age at surgery, type of surgical procedure, and surgery duration were independent risk factors of postoperative complications in the development group. Then, the three factors were included in the prediction model of complications after epilepsy surgery for establishing a nomogram. The illustration of the nomogram is as follows: we can obtain the point corresponding to each prediction indicator, the sum of three points is recorded as the total points, and the predicted risk corresponding to the total points is the probability of complications (Figure 5).

**Figure 6:**
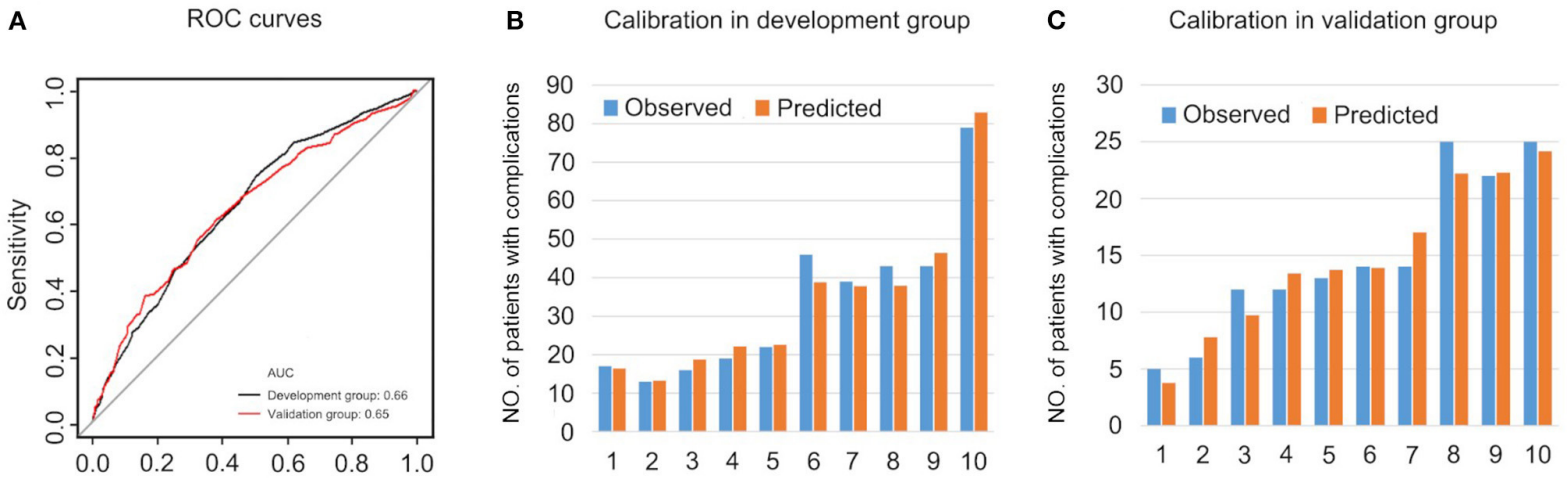
The evaluations of the prediction model. **(A)** ROC curves. **(B)** Calibration in development group. **(C)** Calibration in validation group.

The prediction model is evaluated in Figure 6. The AUC was 0.66 in the development group and 0.65 in the validation group, representing an acceptable discrimination capacity of this model. The Hosmer-Lemeshow test suggests this model has good calibration in the development group (χ^2^ = 4.47, *P* = 0.813) and in the validation group (χ^2^ = 3.12, *P* = 0.927).

## Discussion

In this study, we not only reported the up-to-date complications after epilepsy surgery in the last two decades, but also firstly investigated the risk factors of complications and developed a nomogram for predicting the probability of complications. We found subcutaneous CSF accumulation, neurological deficits, and ICH were the most common complications, and the incidence of severe complications was low. ICH and wound infection were the main severe complications requiring surgical intervention. Seven independent risk factors of postoperative complications and four risk factors of severe complications were identified. Finally, three factors of age at surgery, type of surgical procedure, and surgery duration were included in the prediction model of complications.

### Complications After Epilepsy Surgery

Our study has higher incidence of complications than other large studies (12–15). Currently, there is no consensus on the definition and grading system for complication severity following epilepsy surgery. Thus, the reported complication rate may vary considerably among studies. In some studies, complications were simply classified as either minor or major according to the prognosis (whether it resolved within 3 months) (12, 13, 24). We graded complications based on the therapeutic regimen, so the grades accurately reflected the severity of the complications. For example, ICH requiring surgical evacuation under general anesthesia, which may have been considered minor in the classification system based on time, was considered severe in our study. Another reason is that the percentage of temporal resections in our study is lower than other large studies (43% vs. more than 60%). In the multivariate analysis, we found that temporal resection was the factor decreasing the likelihood of postoperative complications. A 32-year systematic review found that the rate of neurologic deficit after temporal resections was lower than extratemporal resections from 1996 to 2012 (5.1 vs. 19.5%) (17). In addition, subcutaneous CSF accumulation contributed most to the total complication rate in our study. However, subcutaneous CSF accumulation was not reported in other studies. Although the dura was tightly sutured during surgery, CSF in the residual cavity left after resection of the epileptic focus could exude and accumulate under the scalp. Except for subcutaneous CSF accumulation, the incidence of complications in our study was within the range reported in previous studies.

### Risk Factors for Complications

Based on the multivariate analysis, risk factors of complications were confirmed. Being male was a risk factor of complications. Multivariate analysis showed the sex difference among patients with complications compared with no complication and no sex difference among patients with severe complications. Kerezoudis et al. found men were at higher risk for postoperative morbidity than women, which the authors attributed to unmeasured confounding variables (25). The male-to-female ratio was 2:1 in our study. This male proclivity may reflect that men have a stronger desire to achieve seizure control and are more willing to undergo surgery than women.

We found that age at surgery was associated with an increased risk of complications. Compared with a younger cohort, complications following surgery for temporal lobe epilepsy more frequently occurred in individuals aged ≥50 years (26). In two studies, both the rate and severity of complications after epilepsy surgery increased with age (≥35 years) (13, 16). Age was a risk factor for complications in these studies (16, 26). This may be due to the decline in physical condition with increasing age.

In our study, serial number of surgery (smaller number indicating surgery was performed in the earlier period) was related to the risk of complications. This indicated that complications were associated with surgical experience and skill. There was a learning curve for epilepsy surgery (27). A meta-analysis found complication rates decreased markedly over time due to training in surgical procedures and advances in surgical techniques (17). The trend inferred that complication rate declined as the surgeon's experience increased.

We found ICH in infancy increased the risk of postoperative complications. Epileptic foci secondary to ICH were usually extensive. Among patients with ICH in infancy, 66.7% underwent multilobar resections and 46.2% underwent extratemporal resections in our study. Extensive resections increased the possibility of injuring the eloquent cortex and its tracts. Compared with temporal resections, both overall and persistent neurological complications more frequently occurred in extratemporal and/or multilobar resections (17). Therefore, temporal resections were safer than other types of surgery. Moreover, extensive resections prolonged the surgery duration. Our study showed that surgery duration was associated with complications. Golebiowski et al. reported surgical duration as an independent risk factor for medical complications after brain tumor surgery (28). Complication rates were highest in patients with operations lasting ≥6 h (28).

Complications more frequently occurred in patients with APOS in our study. The relationship between seizures and complications were mutual. APOS was common after epilepsy surgery (23, 29) and was attributed to transient perioperative factors, such as cortical irritation, brain edema, hemorrhage, and low concentrations of antiepileptic drugs (23). Conversely, seizures have adverse effects on patient's post-surgery recovery (29). Particularly, status epilepticus is a life-threatening seizure with a high mortality rate (30). Two patients in our study died of frequent APOS. Thus, APOS may be a sign of the need for increased patient monitoring.

Tumors were a risk factor for severe complications, in line with the literature. In our study, tumors were diagnosed in 25 out of 82 (30.5%) patients with severe complications. Neurological deficits (18 patients) and ICH (5 patients) were the most common severe complications in patients with tumors. Tumoral surgery has high mortality and a high complication rate (31, 32). Mortality following brain tumor surgery reported in the literature was more than 2%.

### Prediction Model of Complications

We developed a prediction model of complications. This model demonstrated that age at surgery, type of surgical procedure, and surgery duration were the main risk factors for individualized prediction of the probability of complications. Previous studies only reported the rate of complications in whole patients with epilepsy surgery (13–15). However, for any individual patient undergoing surgical therapy, the probability of complications was not prescient. Thus, the prediction model is useful for surgeons to identify patients with a high risk of complications and enhance the postoperative management of these patients for decreasing and avoiding the incidence of complications. The nomogram also relieves patients' fear associated with the risks of surgical procedures.

### Strengths and Limitations

Our study has several strengths. First, the surgical procedures in our study were performed by a single surgeon at a single institution. Thus, the surgical environment, surgical strategy, and care protocols for patients undergoing epilepsy surgery were consistent. Our study may more accurately reflect the real-world side effects of epilepsy surgery. Second, our study firstly identified the risk factors for complications based on the large-scale sample. Third, we firstly established the individualized prediction model of complications, not reported in previous studies.

The present study has several limitations. The study design was retrospective, causing potential biases. Furthermore, our study was limited to a 3-month postoperative period and therefore could not provide insight into long-term outcomes. In future, long-term postoperative complications need to be analyzed. Finally, the discrimination and calibration of the prediction model are not perfect. Therefore, a prospective multicenter study is needed to improve the prediction model. Although these limitations exist, our study remains valuable for the up-to-date overview of complications, improvement in patient management, and understanding in surgical procedures.

## Conclusions

We describe postoperative complications of epilepsy surgery and analyze the associated risk factors. Our findings reinforce the safety of epilepsy surgical procedures. Most complications are reversible. Severe complications occurr at a low rate. Risk factors for complications are identified. The prediction model is a beneficial supplementary tool for clinical practice.

## Data Availability Statement

The original contributions presented in the study are included in the article/supplementary material, further inquiries can be directed to the corresponding author/s.

## Ethics Statement

The studies involving human participants were reviewed and approved by Ethical Committee of Xi'an Jiaotong University Medical College First Affiliated Hospital. Written informed consent to participate in this study was provided by the participants' legal guardian/next of kin.

## Author Contributions

HZ has full access to all of the data in the study, takes responsibility for the integrity of the data, and the accuracy of the data analysis. QM, HW, and HZ: study concept and design. YL, HL, and SD: acquisition, analysis, or interpretation of data. YL, HW, and HZ: drafting of the manuscript. YL, HW, and HL: statistical analysis. QM and HZ: study supervision. CD, KL, and XL: follow-up with patients. All authors: critical revision of the manuscript for important intellectual content.

## Funding

This research was supported by the Natural Science Basic Research Program of Shaanxi (Program Nos. 2021SF-083 and 2021JQ-384), the Innovation Capability Support Program of Shaanxi (Program No. 2021LCZX-01), the Institutional Foundation of The First Affiliated Hospital of Xi'an Jiaotong University (No. 2020ZYTS-01), and the National Natural Science Foundation of China (Nos. 81471322 and 81601132).

## Conflict of Interest

The authors declare that the research was conducted in the absence of any commercial or financial relationships that could be construed as a potential conflict of interest.

## Publisher's Note

All claims expressed in this article are solely those of the authors and do not necessarily represent those of their affiliated organizations, or those of the publisher, the editors and the reviewers. Any product that may be evaluated in this article, or claim that may be made by its manufacturer, is not guaranteed or endorsed by the publisher.
